# Surgical removal of unexpected oral squamous cell carcinoma arising from a maxillary odontogenic keratocyst in a young female patient: a case report and literature review

**DOI:** 10.1186/s12903-025-07562-2

**Published:** 2026-01-15

**Authors:** Tasneem A. Amer, Mai M. Saleh, Mohammed S. Alalfy

**Affiliations:** 1 Lecturer Oral and Maxillofacial Surgery, Faculty of Dentistry, Alexandria University, Alexandria, Egypt; 2 Lecturer Oral Pathology, Faculty of Dentistry, Alexandria University, Alexandria, Egypt; 3 Lecturer of Diagnostic and Interventional radiology, Kafrelsheikh University, Kafrelsheikh, Egypt

**Keywords:** Squamous cell carcinoma, Odontogenic keratocyst, Maxillectomy

## Abstract

**Background:**

Although odontogenic cysts in the maxilla are often seen, malignant changes inside these cysts are rather uncommon. According to reports, the occurrence of carcinomas developing in odontogenic cysts ranges from 1 to 2 per 1,000. Squamous cell carcinoma (SCC) arising from an odontogenic keratocyst (OKC) is a rare malignant transformation that typically presents in the jaws. This form of carcinoma is locally aggressive and often associated with a poor prognosis, especially when the diagnosis is delayed.

**Case presentation:**

This report discusses a healthy 13-year-old girl with no medical, dental, or surgical background who visited the Oral and Maxillofacial Surgery Department due to mild, dull discomfort and gradually increasing swelling in the right maxilla, originating from the palatal side for the past six months. The surgical procedure was conducted under general anesthesia during which a biopsy was collected from the patient; subsequent processing involved haematoxylin and eosin (H&E) staining and examination under a light microscope. The diagnosis of the lesion was made as an invasive, moderately differentiated, keratinizing SCC arising within an odontogenic keratocyst. The patient underwent a subtotal maxillectomy. An obturator was placed postoperatively to restore function and aesthetics.

**Conclusion:**

SCC arising from an OKC is an exceptionally rare and aggressive entity, particularly in pediatric patients. This case highlights the diagnostic challenge posed by seemingly benign cystic lesions and emphasizes the importance of histopathological evaluation of all excised jaw cysts.

## Background

It is quite uncommon for cancer to develop in the bone. “A squamous cell carcinoma (SCC) that occurs in the jaws without any first communication to the oral mucous membrane.” is the definition of a primary intraosseous squamous cell carcinoma (PIOSCC) [1].

The World Health Organisation (WHO) proposed the name primary intraosseous carcinoma (PIOC) in 1972 and categorised this lesion as an odontogenic carcinoma since the epithelium involved in odontogenesis is the source of this lesion [2]. It is possible for PIOC to develop de novo from suspected odontogenic cell remains (such as reduced enamel epithelium) or from the lining of an odontogenic cyst [3].

The lining epithelium of odontogenic cysts may experience cystic expansion, keratinization, or undergo dysplasia. The more frequently occurring tumors that develop from the lining of odontogenic cysts include benign odontogenic tumors such as odontoma, Pindborg tumor, and adenomatoid odontogenic tumor [4]. However, it is well-established that the lining epithelium of an odontogenic cyst has the potential to change into a SCC or a mucoepidermoid carcinoma [3].

The clinical and radiographic characteristics of these two entities are often comparable to those of benign, expansive, central odontogenic tumors or jaw cysts. Paraesthesia rarely occurs. Radiographically, they resemble the cystic lesions from which they originated [5].

Carcinomatous transformation exists in less than 1% of the odontogenic cysts. Approximately 80% of SCCs are identified after the age of forty, with a 2:1 male to female proportion. Most of the lesions are moderate to well-differentiated carcinomas, and metastasis from those lesions has not occurred very often [6].

In 1913, Loos first described PIOSCC as a central epidermoid carcinoma of the jaw [7]. It was changed to an interalveolar epidermoid carcinoma by Wills in 1948 [8]. In 1969, Shear called it primary intra-alveolar epidermoid carcinoma [9]. The lesion was classified as an odontogenic carcinoma by the WHO and Pindborg in 1972, when they accepted the name PIOSCC [10]. Elzay, after that, changed the WHO classification for the jaw’s PIOC [11]. In 1948, Slootweg and Müller made minor modifications to Elazy’s classification by taking into account several potential etiological factors [12]. Intraosseous mucoepidermoid carcinoma was added as a fourth entity of PIOCs by Waldron and Mustoe to complete the classification [13]. Ultimately, PIOSCCs were reclassified as: first, a solid tumor that leads to bone resorption, second, SCC developing from an OKC lining or carcinoma developing in other odontogenic cysts, and third, SCC associated with benign epithelial odontogenic tumors in the (2022 )WHO classification, which replaced the previous terms [14].

Primary intraosseous carcinomas arising from the wall of an OKC are a rare tumor occurring within the jaw bones. A study by Bodner et al. in 2011 conducted a retrospective study of 116 cases of PIOC between 1938 and 2010. The result of this work showed that there have been only sixteen cases of PIOC developing from keratocyst, accounting for 14% of all the odontogenic cysts [15].

An impacted tooth is more than just a dormant thing. It is a major pathological risk factor that can lead to jaw changes that are both transformational and frequently destructive. These alterations include severe, bone-destroying cysts, the loss of nearby normal teeth, and common infections like pericoronitis and even oral and maxillofacial cysts or tumors. It has been found that cystic or neoplastic lesions arise in proximity to the impacted tooth in 16% of cases, most commonly during the second and third decades of life [16]. To avoid these issues, early detection and treatment of impacted teeth depend on routine dental monitoring along with radiographic examination [17].

The occurrence of carcinomatous transformation in odontogenic cysts has been documented to range from 0.13% to 3% [18]. There is an ongoing debate regarding which type of odontogenic cyst carries the highest risk of malignant transformation. Follicular (non-inflammatory) cysts have been reported as the most common etiology by Yasuoko [19] and Borras-Ferreres [18] whereas, Jain [20] and Bodner [15] have identified radicular (inflammatory) cysts as the most frequently undergoing transformation.

The mechanisms underlying the malignant transformation of odontogenic cysts remain largely unclear. Nevertheless, it is believed that carcinogenesis induced by chronic inflammation from persistent lesions may represent one possible pathway. Chronic inflammation has the potential to damage DNA, proteins, and gene expression, which can consequently result in genetic mutations that increase the risk of malignant transformation. Thus, it has been proposed that the likelihood of malignant conversion of the epithelial lining in an odontogenic cyst is significantly reduced in the absence of inflammation. Moreover, odontogenic cysts that exhibit keratinization are at a higher risk for malignant alterations compared to their nonkeratinizing counterparts. Reports indicate that the malignant transformation of the lining of odontogenic keratocysts constitutes 14% of all malignant transformations associated with odontogenic cysts [15].

The management of OKC varies significantly. It encompasses conservative surgical procedures such as marsupialization, decompression, and irrigation, which are often paired with distant definitive treatment. The definitive management of primary OKC involves enucleation and curettage and may also incorporate cryotherapy or chemoablation. Reports indicate that conservative treatments, commonly utilized for larger lesions, may carry an increased risk of malignant transformation of the cyst lining during the interval from diagnosis to definitive excision [21].

There are a few documented cases of malignant transformation arising from OKCs. Peng ye et al. investigated the clinicopathologic features of PIOSCC arising from an OKC (PIOSCC ex OKC) at Peking University School and Hospital of Stomatology. They also conducted a systematic review of studies on PIOSCC ex OKC by using online databases from their inception until February 2020. They found that all lesions were located in the mandible, which is consistent with the predilection site of OKCs [22]. In contrast, some studies showed the occurrence of SCC in maxillary odontogenic keratocysts [23, 24]. 

Phase I of treatment for most of these cases was either enucleation or incisional biopsy. Following an unanticipated discovery of carcinoma, additional treatment consisted of radical resection in the majority of cases, neck dissection and radiation, or chemotherapy in 1/3 of cases [25].

## Case presentation

In this case report, we present a case of an OKC that was found to have undergone malignant transformation into SCC. A 13-year-old female patient without relevant medical history complains of a mild, dull pain and slowly growing swelling in the right maxilla from the palatal aspect for six months.

**Fig. 1:**
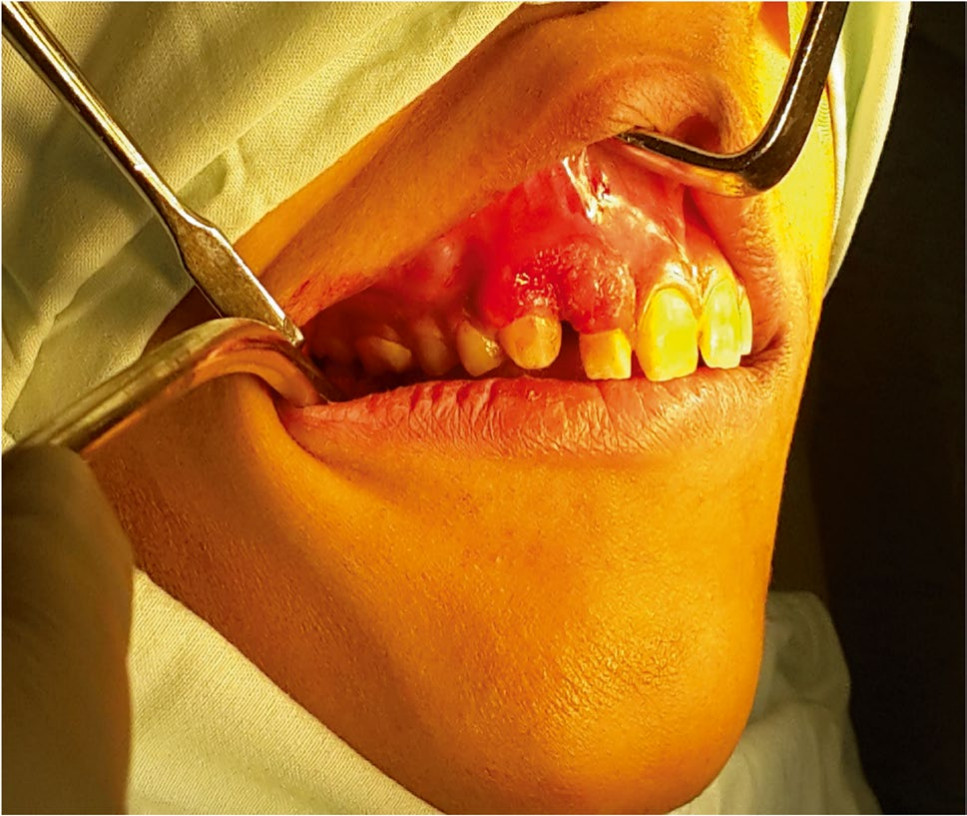
Patient presented with a localized swelling in the right maxilla

A few months prior to examination, the patient noticed a gradually increasing swelling on the palatal aspect of the right maxilla, despite otherwise appearing clinically normal. Intraoral examination revealed a localized swelling extending from the upper right central incisor to the upper right second premolar. The buccal mucosa appeared normal, except in the region of the right lateral incisor, where mucosal erosion was noted (Fig. 1). The palatal mucosa showed secondary inflammation, with evidence of palatal bone perforation upon palpation. Only the upper lateral incisor was mobile, while the other teeth were not sensitive to percussion or mobile. There were no reports or observations of paraesthesia, anaesthesia, nasal obstruction, or visual disturbance. Cervical lymphadenopathy was absent.

**Fig. 2:**
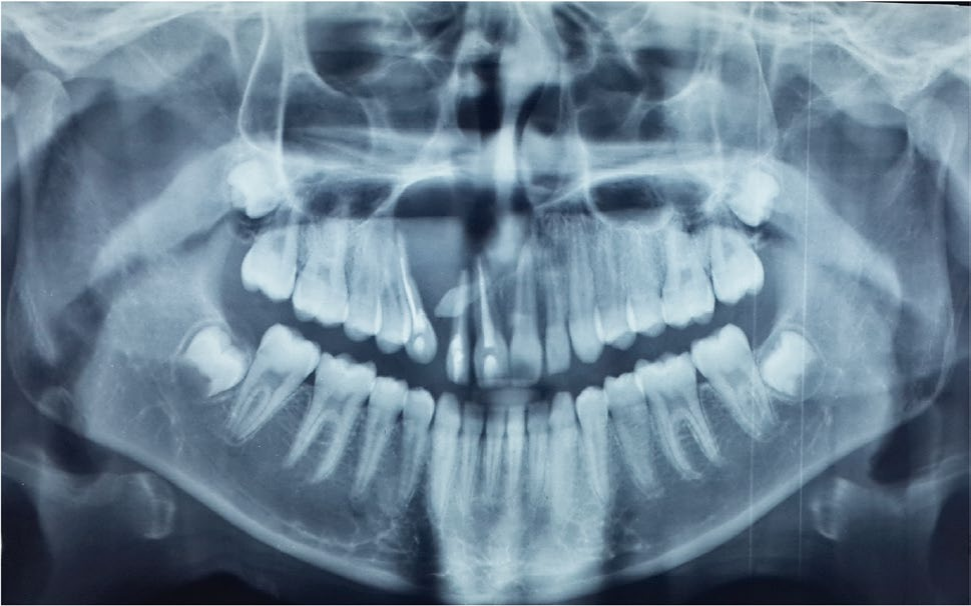
Panoramic x-ray showing a well-defined radiolucency with related supernumerary tooth, extended from the upper left central incisor to the upper right canine

Panoramic radiograph revealed a well-defined unilocular radiolucency related to a supernumerary tooth, extending from the upper left central incisor to the upper right canine, with displacement of the canine root and no evidence of additional pathology (Fig. 2).

Based on the patient’s history, clinical findings, and radiographic features—particularly the association with an impacted tooth—the lesion was initially strongly suspected to be a dentigerous cyst.

**Fig. 3:**
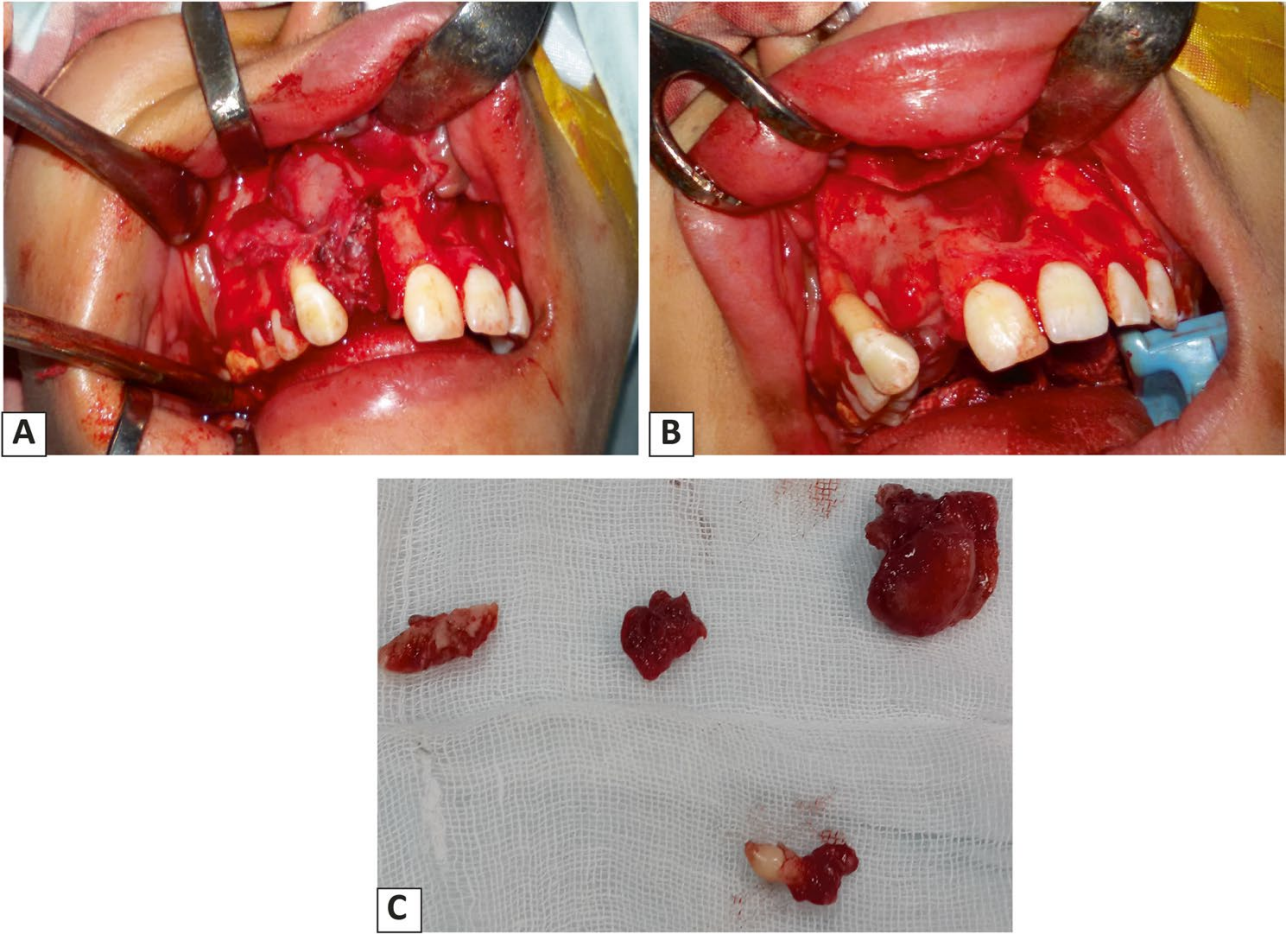
**A**: reflection of periosteal flap showing the cystic lesion, **B**: total excision of the lesion showing the boney cavity after removal of the cystic lesion, **C**: excisional biopsy of the cystic lesion with extraction of supernumerary tooth and related upper right lateral incisor

The surgical procedure was performed under general anaesthesia. A full-thickness muco-periosteal flap was performed using blade no. 15, extending one tooth anterior and one tooth posterior to the cystic cavity, then the muco-periosteal flap was reflected using a periosteal elevator to expose the bone. The overlying thin shell of bone was removed to expose the cystic lesion, which was enucleated with the involved tooth; the lateral incisor was also extracted, and apicectomy was performed on the central incisor (Fig. 3). The surgical wound was closed using suture 3 − 0. Then the excised lesion was sent for histopathological examination.

**Fig. 4:**
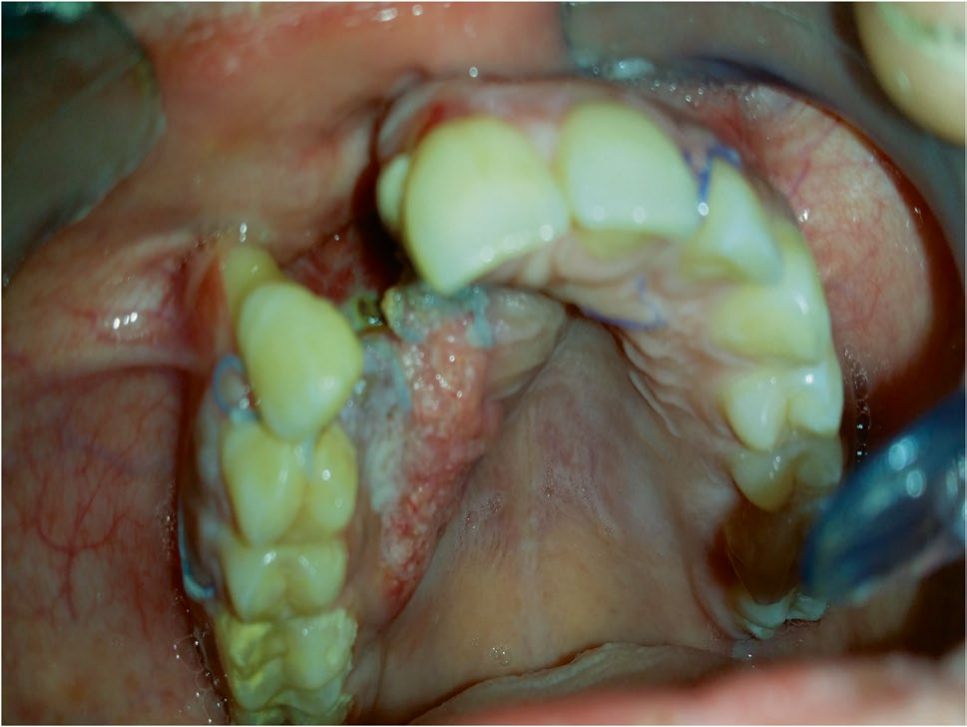
Photograph of the surgical site 2 weeks postoperative without soft tissue healing. Instead, soft tissue thickening was observed, along with irregular tissue proliferation extending across the palatal mucosa

Approximately two weeks after the initial surgery and before the pathology report, there was no evidence of soft tissue healing at the surgical site, including the area of the extracted right lateral incisor. Instead, soft tissue thickening was observed, along with irregular tissue proliferation extending across the palatine mucosa (Fig. 4). These findings raised concern for residual pathology or possible malignant transformation,

The biopsy sample was fixed in 10% neutral buffered formalin, then processed and embedded in paraffin wax using the conventional procedures. Serial Sects. 3–4 μm thick were placed on glass slides and stained using hematoxylin and eosin (H&E). Then, the slides were studied under light microscopy.

**Fig. 5:**
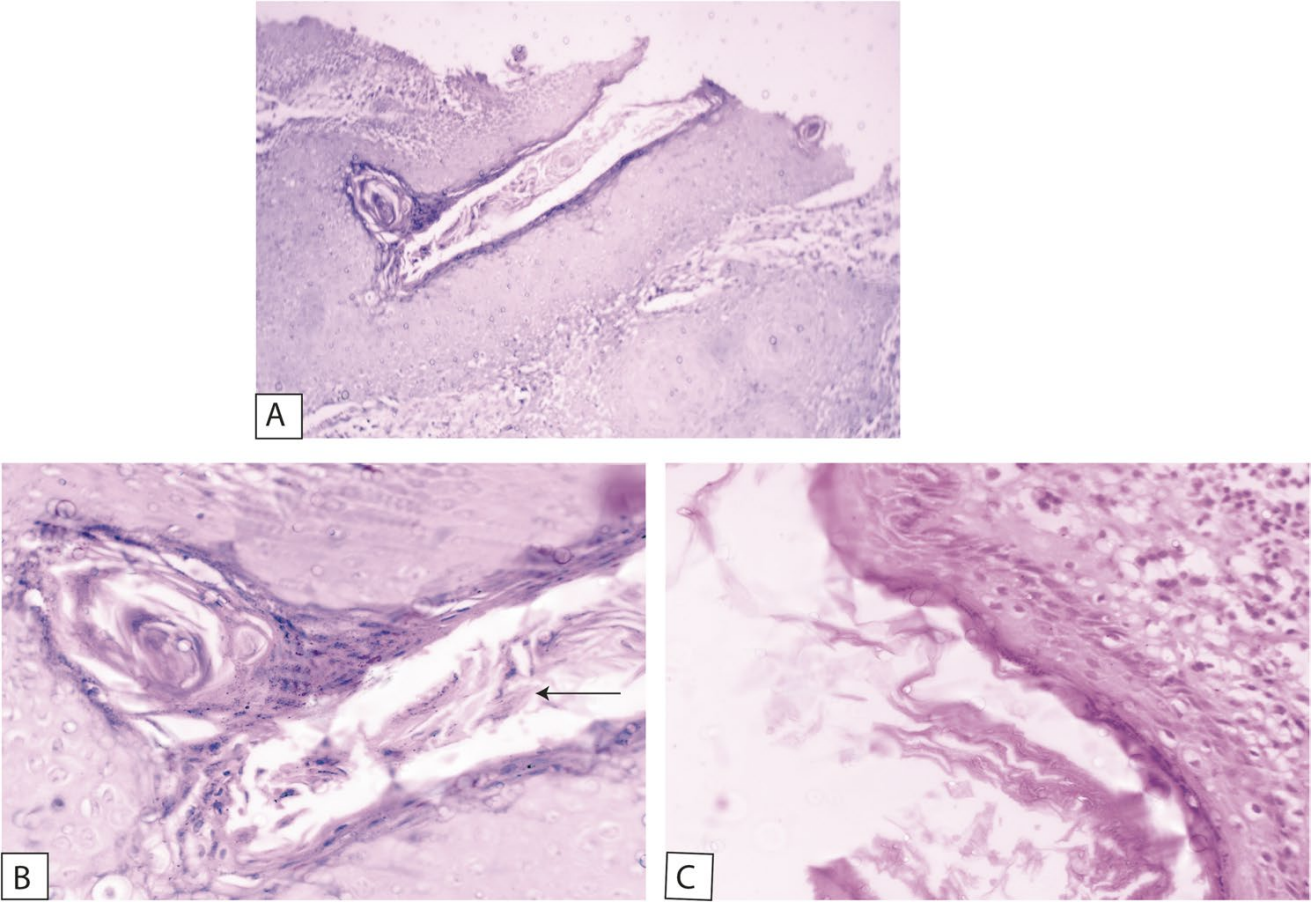
**A** Photomicrograph of odontogenic keratocysts showing hyperplastic corrugated parakeratinized epithelial lining (H&E stain, x100). **B** High power of previous image showing the lumen of the cyst with large amount of keratin (arrow) and surrounded by epithelial lining which is parakeratinized (H&E stain, x400). **C** Photomicrograph showing epithelial lining with palisading basal cells and corrugated epithelial lining (H&E stain, x400)

**Fig. 6:**
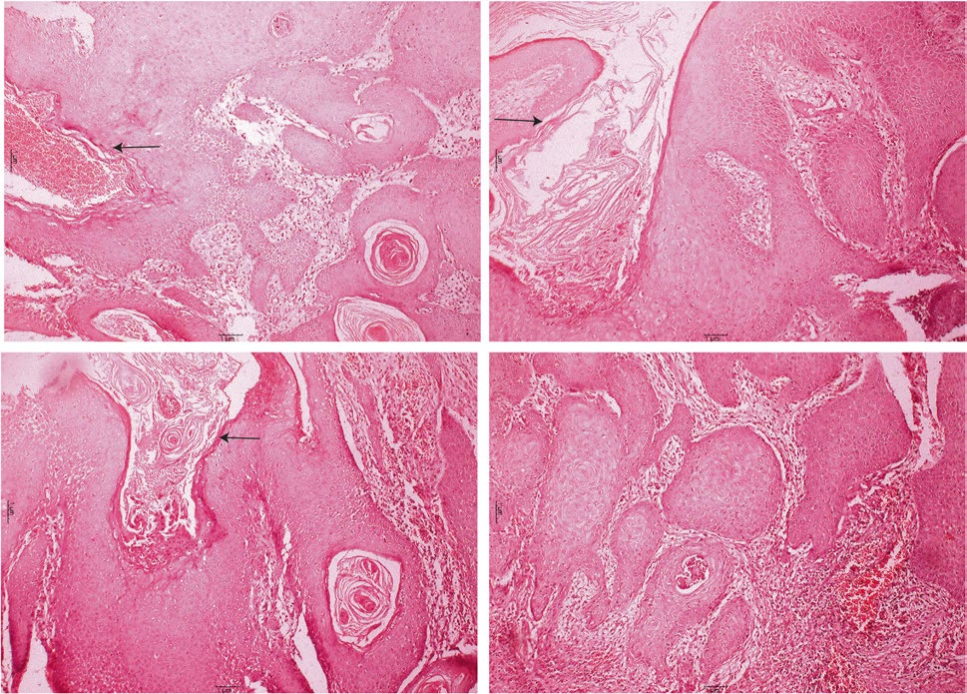
Photomicrograph showing a cystic cavity lined by parakeratinized stratified squamous epithelium (black arrows). Certain areas of the epithelial odontogenic surface undergo unusual squamous metaplasia with evident malignant changes in the underlying fibrous capsule of the detected keratocyst. Epithelial pearls with central keratin formation and malignant cell nests are detected (H&Ex100)

**Fig. 7:**
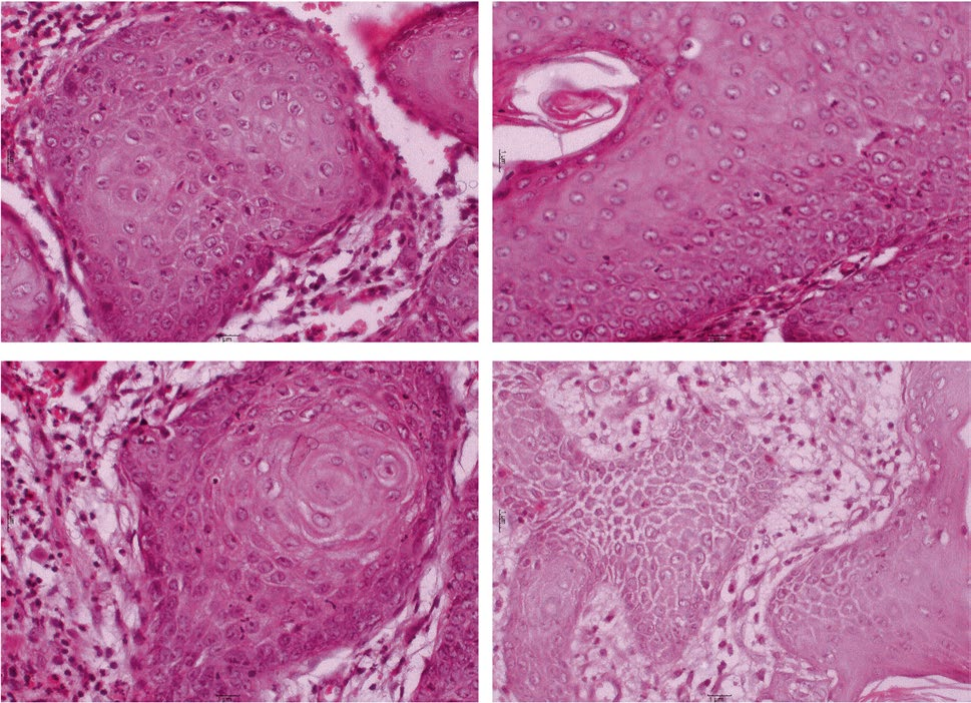
Photomicrograph revealed epithelial pearls and cell nests of SCC with malignant criteria such as pleomorphism, hyperchromatism and normal & abnormal mitotic figures (H&Ex400)

The histological examination demonstrated a cystic cavity lined by corrugated parakeratinized stratified squamous epithelium with intraluminal keratin. Some areas showed partially uniform thickness with palisaded basal cells. The supra-basal cells are polyhedral and show intercellular edema and intercellular bridges. The epithelium-connective tissue interface is flat with an absence of rete pegs. Certain areas of the epithelial odontogenic surface undergo unusual squamous metaplasia with evident malignant changes in the underlying fibrous connective tissue capsule of the detected keratocyst. Epithelial pearls with central keratin formation and malignant cell nests are detected. Malignant criteria are detected, such as pleomorphism, hyperchromatism, as well as normal & abnormal mitotic figures. So, the lesion was diagnosed as an invasive, moderately differentiated, keratinizing SCC developing in an OKC (Figs. 5, 6 and 7).

**Fig. 8:**
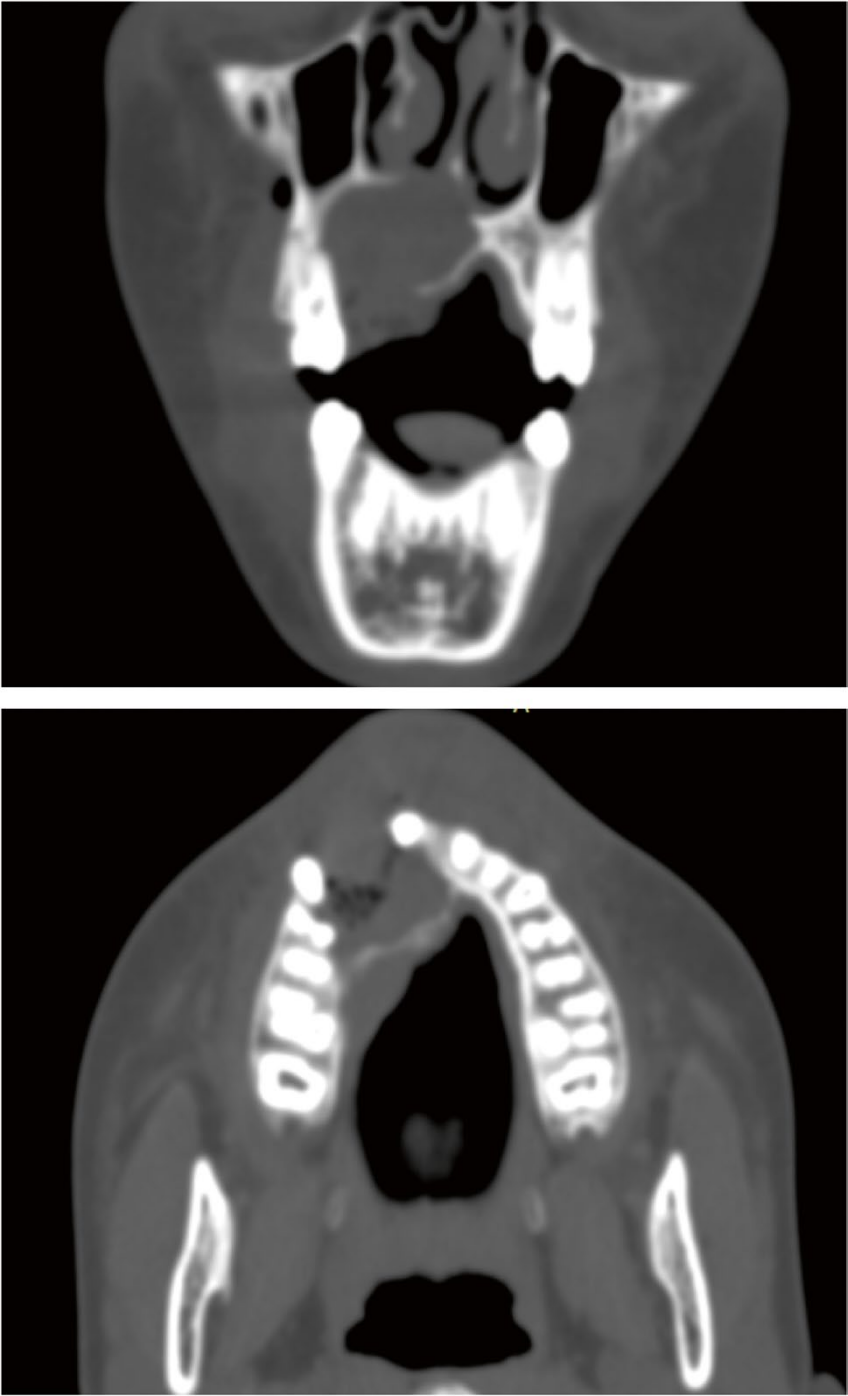
CT scan coronal and axial cuts showing bony defect after removal of cystic lesion and palatal thickening

**Fig. 9:**
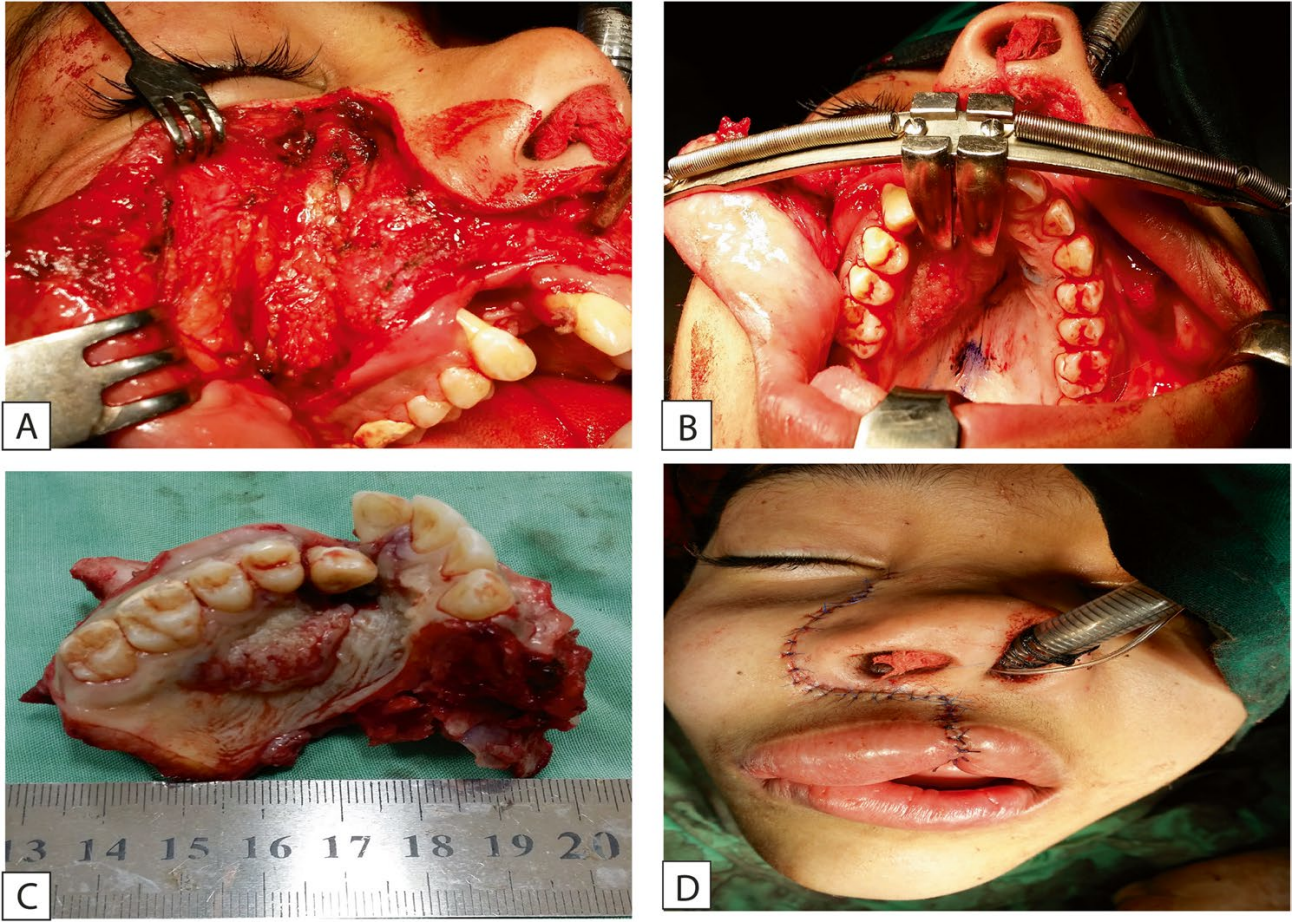
**A**, **B**: Weber-Ferguson incision, providing adequate access for subtotal maxillectomy. **C**: Excised maxilla including SCC with safety margins. **D**: incision was closed meticulously to ensure optimal healing

**Fig. 10:**
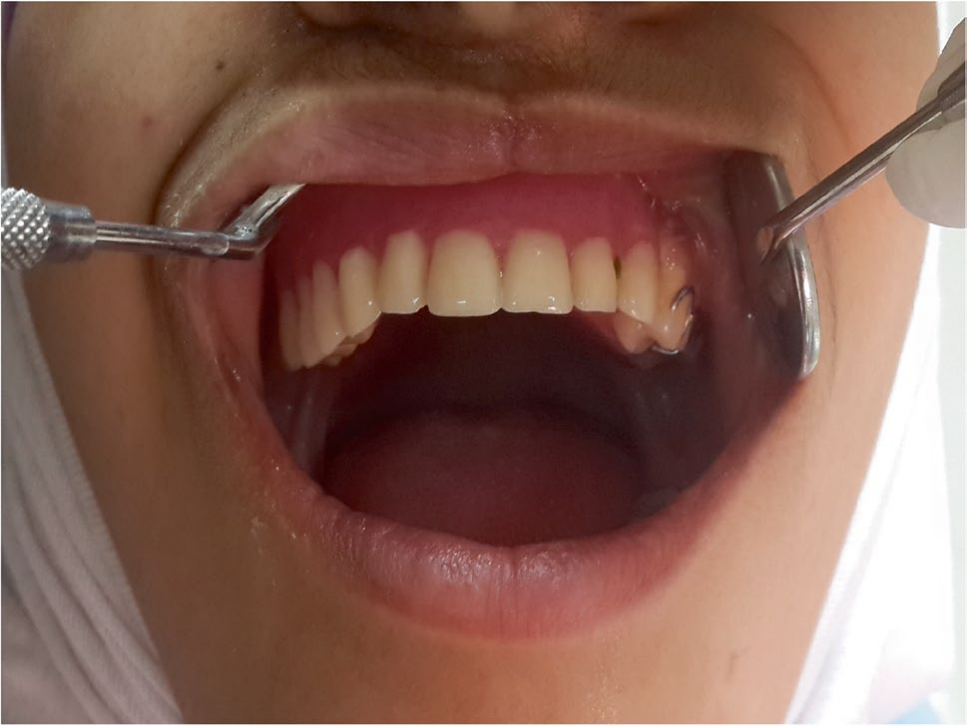
photograph showing patient follow up after subtotal maxillectomy with obturator perfectly replacing the defect and restoring esthetics and function

The patient subsequently underwent a comprehensive workup, including a head and neck computed tomography (CT) scan (Fig. 8) and abdominal and pelvic ultrasound to exclude regional and distant metastases. Based on the clinical and radiographic findings, and after consultation with an oncologist, the patient was referred for a subtotal maxillectomy for total excision of the pathological tissue with enough safety margins. A subtotal maxillectomy was performed via a Weber-Ferguson incision, providing adequate access for complete surgical excision of the lesion (Fig. 9-A, B, C). The incision was closed meticulously to ensure optimal healing and achieve a more aesthetically acceptable outcome, particularly considering the young female patient’s age and appearance (Fig. 9-D). Histopathological analysis of the resected specimen confirmed clear surgical margins. A maxillary obturator was fabricated and delivered postoperatively to support function and aesthetics (Fig. 10). The patient was kept under regular follow-up for six months after the second surgery, during which no clinical signs of recurrence were detected. Unfortunately, the patient was lost to follow-up beyond this period.

### Discussion and conclusions

Odontogenic keratocyst (OKC) is a benign developmental cyst of odontogenic origin, recognized for its locally aggressive behavior and tendency for recurrence. Reported recurrence rates vary widely, from 0% to 62%, making it a clinically significant and challenging entity in maxillofacial pathology [26].

Malignant changes of the OKC lining are uncommon, despite its high recurrence rates and locally destructive behavior. Rare cases of malignant transformation into SCC have been reported [27]. The clinical and histological characteristics of a destructive maxillary cyst exhibiting moderately differentiated SCC were reported in this case report.

It’s interesting to note that the full histopathological analysis of the biopsies showed foci with nests of SCC emerging from the OKC lining that showed different levels of dysplasia. Prior to a diagnosis of a carcinoma arising in an odontogenic cyst, it is important to eliminate secondary involvement of a cyst by an unrelated adjacent carcinoma or cystic degeneration in a primary intraosseous carcinoma or in a metastatic deposit [28].

Gardner listed specific features for diagnosing SCC that arises in an odontogenic cyst: the lack of carcinomatous alterations in the lining epithelium, the lack of a source of carcinoma in the surrounding tissues, and the microscopic transition zone from benign cystic lining epithelium to invasive squamous cell carcinoma [29].

Waldron introduced a fourth criterion. Physical, radiological, and subsequent clinical examinations are necessary to rule out the potential that the lesion is a distant tumor metastasis [30].

Symptoms consistent with an odontogenic cyst were evident in the patient presented in this report. Large, untreated odontogenic cysts are known to cause thinning of the cortical bone and, in some cases, perforation of the palatal aspect of the maxilla. In this case, the mobility of the adjacent tooth and the presence of a supernumerary tooth within the cystic lesion supported the diagnosis of an odontogenic cyst, particularly one resembling a dentigerous cyst. Additionally, the patient’s age was within the typical range for the development of dentigerous cysts. This was in agreement with the literature regarding odontogenic manifestations [31].

In our case, after cystic lesion enucleation in the 1st surgery, the transformation was suspected clinically even before histopathology was available, based on delayed mucosal healing and abnormal proliferation on the palatal mucosa. This emphasizes the importance of close clinical follow-up during the early postoperative phase, especially in lesions initially thought to be benign.

Before initiating timely surgical intervention in such cases, a thorough understanding of the tumor’s biological behavior and cellular kinetics is essential. Since its initial description, the OKC is considered one of the most controversial pathological lesions of the maxillofacial area [32]. Although previously classified as a benign neoplasm in the 2005 WHO classification, OKC was reclassified as a cystic lesion in the 2017 and remains so in the 2022 WHO classification of Head and Neck Tumours [14]. On rare occasions, though, the OKC epithelial lining exhibits signs of dysplasia and malignant transformation to SCC [33].

Odontogenic tumors, which make up only 1% of all oral tumors, are classified as rare tumors in the most recent edition of the WHO. They are also benign entities that have a high recurrence rate and may behave aggressively [34]. In the 2017 edition, keratocystic odontogenic tumor is nowadays classified as a cyst and called an OKC [35].

Since carcinomatous transformation occurs in only about 1% of all odontogenic cysts, these carcinomas often present with clinical features that closely resemble those of benign expansile jaw lesions, not associated with pain or paresthesia. On plain radiographs, they appear similar to cystic lesions from which they arise. Therefore, depending on plain panoramic radiography in this case, which suggested a well-defined cystic lesion, was considered a limitation. While useful for preliminary evaluation, plain radiographs may underestimate the lesion’s true extent and fail to reveal cortical perforation or soft tissue involvement. CT would have provided more detailed information regarding the lesion’s boundaries, aggressiveness, and possible invasion of adjacent structures. Marcelo Cavalcanti et al. have claimed that because a CT scan provides a greater understanding of the damage to surrounding structures, it could be a more sensitive method of identifying a malignant change in the lesions [36].

According to previous reports, PIOSCC predominantly affects the mandible approximately 79% compared with the maxilla 21% [1, 15]. Cases of PIOSCC arising from an OKC have been documented across a broad age spectrum, with a mean age of 57 years and a male-to-female ratio of 2:1. Similar findings were observed in cases reported by Aboul-hosn Centenero et al.. in 2006 [37], Mosqueda Taylor et al.. in 2003 [38] and Scheer et al.. in 2004 [39]. However, this age distribution contrasts with our reported case, in which the patient was only 13 years old, representing an exceptionally early onset for such malignant transformation.

To date, only a few pediatric cases of SCC arising from odontogenic cysts have been published. Ours may be among the youngest reported cases, reinforcing the need to consider malignant potential even in the pediatric population, where OKC is more often associated with syndromic or developmental origins [40]. Other published studies showed SCC arising from maxillary OKC were reported in older age [23, 41]. 

The surgical approach via Weber-Ferguson incision allowed for adequate access and complete excision with safety margins. This technique is well-documented in the management of maxillary tumors and provides both functional and aesthetic advantages when carefully closed, particularly in young patients [42]. Postoperative rehabilitation with a maxillary obturator proved effective in restoring speech, mastication, and aesthetics.

Although the patient was recurrence-free for six months postoperatively, she was unfortunately lost to follow-up thereafter. Long-term follow-up is essential given the potential for delayed recurrence, particularly in malignancies arising within odontogenic cysts. Literature suggests at least five years of follow-up with periodic imaging and clinical evaluation to monitor for recurrence or metastasis [34].

The histopathological examination in this case revealed carcinomatous alterations in the lining epithelium of the odontogenic keratocyst. The occurrence of carcinoma associated with this OKC was unforeseen, paralleling other documented instances where similar unexpected features were noted [28].

There were no indications of nerve paraesthesia or pain, likely due to the malignant changes still in the early stages of development. This may account for the well-defined radiolucency observed in the panoramic film. This contrasts with the observations suggesting that malignant transformations in odontogenic cysts had to be suspected if the radiolucent area exhibits uneven edges with indentations and poorly defined borders [36], a scenario not applicable to the current report.

Instances of the epithelial lining of OKC exhibiting dysplastic criteria and carcinomatous transformation are seldom reported, making this particular case significant due to the presence of malignant changes, especially considering that it involves a pediatric patient [43].

In conclusion, the clinical manifestations, radiographic, and histopathologic criteria of a carcinomatous change of an OKC in the maxilla of a young female patient are presented in this unique case report of moderately differentiated SCC. It also emphasizes how crucial it is to carefully examine any removed pathologic tissue histopathologically due to the potential for carcinomatous change in the cystic lesion’s epithelial lining.

## Data Availability

The datasets used during the current study are available from the corresponding author on reasonable request.

## Abbreviations

SCC: Squamous cell carcinoma
OKC: Odontogenic Keratocyst
H&E: Haematoxylin and Eosin
PIOSCC: Primary intraosseous squamous cell carcinoma
WHO: World Health Organisation
PIOC: Primary intraosseous odontogenic carcinoma
CT: Computed tomography

## References

[1] Tamgadge S, Tamgadge A, Modak N, Bhalerao S. Primary intraosseous squamous cell carcinoma arising from an odontogenic keratocyst: a case report and literature review. Ecancermedicalscience. 2013;7:316. 23717337 10.3332/ecancer.2013.316 PMC3660158

[2] Barnes L, Eveson JW, Reichart PSD, editors. World health organization classification of tumors: pathology and genetics of head and neck tumors. Volume 290. Lyon: JARC; 2005.

[3] Saxena C, Aggarwal P, Wadhwan V, Bansal V. Primary intraosseous squamous cell carcinoma in odontogenic keratocyst: a rare entity. J Oral Maxillofac Pathol: JOMFP. India. 2015;19:406. https://pubmed.ncbi.nlm.nih.gov/26980976/10.4103/0973-029X.174615. 10.4103/0973-029X.174615 PMC4774301 26980976

[4] Maurette PE, Jorge J, de Moraes M. Conservative treatment protocol of odontogenic keratocyst: a preliminary study. J Oral Maxillofac Surg. 2006;64(3):379–83. 16487797 10.1016/j.joms.2005.11.007

[5] Mohanty S, Bansal N, Verma A, Urs AB. Mandibular primary intraosseous carcinoma arising from long-standing odontogenic keratocyst. Oral Surg Oral Med Oral Pathol Oral Radiol. United States. 2024;137:e8–15. 10.1016/j.oooo.2023.07.012. 10.1016/j.oooo.2023.07.012 38155014

[6] Maria A, Sharma Y, Chhabria A. Squamous cell carcinoma in a maxillary odontogenic keratocyst: a rare entity. Natl J maxillofac Surg. India; 2011;2:214–8. 10.4103/0975-5950.94486. 10.4103/0975-5950.94486 PMC3343400 22639518

[7] Loos D. Central epidermoid carcinoma of the jaws. Dtsch Mscnr Zahnhelic. 1913;31:308 10. Quoted in: Morrison R, Deeley TJ. Intra alveolar carcinoma of the jaw. Treatment by supervoltage radiotherapy. Br J Radiol. 35:321–6. 10.1259/0007-1285-35-413-321. 10.1259/0007-1285-35-413-321 14476303

[8] Wills RA. Pathology of Tumors. London, England :CV Mosby Co; 1948. p. 310–6. 10.4103/0973-029X.174615.

[9] Shear M. Primary intra-alveolar epidermoid carcinoma of the jaw. J Pathol. 1969;97(4):645–51. 5354042 10.1002/path.1710970409

[10] Kramer IR, Pindborg JJ, Shear M. The WHO histological typing of odontogenic Tumours. A commentary on the second edition. Cancer. 1992;70(12):2988–94. 1451083 10.1002/1097-0142(19921215)70:12<2988::aid-cncr2820701242>3.0.co;2-v

[11] Elzay RP. Primary intraosseous carcinoma of the jaws. Review and update of odontogenic carcinomas. Oral Surg Oral Med Oral Pathol. 1982;54(3):299–303. 6957827 10.1016/0030-4220(82)90099-8

[12] Slootweg PJ, Müller H. Malignant ameloblastoma or ameloblastic carcinoma. Oral Surg Oral Med Oral Pathol. 1984;57(2):168–76. 6366686 10.1016/0030-4220(84)90207-x

[13] Waldron CA, Mustoe TA. Primary intraosseous carcinoma of the mandible with probable origin in an odontogenic cyst. Oral Surg Oral Med Oral Pathol. 1989;67(6):716–24. 2662106 10.1016/0030-4220(89)90014-5

[14] Soluk-Tekkesin M, Wright JM. The world health organization classification of odontogenic lesions: A summary of the changes of the 2022 (5th) edition. Turk Patoloji Derg. 2022;38(2):168–84. 35578902 10.5146/tjpath.2022.01573 PMC9999699

[15] Bodner L, Manor E, Shear M, van der Waal I. Primary intraosseous squamous cell carcinoma arising in an odontogenic cyst: a clinicopathologic analysis of 116 reported cases. J Oral Pathol Med. 2011;40(10):733–8. 21689161 10.1111/j.1600-0714.2011.01058.x

[16] Kruger E, Thomson WM, Konthasinghe P. Third molar outcomes from age 18 to 26: findings from a population-based new Zealand longitudinal study. Oral Surg Oral Med Oral Pathol Oral Radiol Endod. 2001;92(2):150–5. 11505260 10.1067/moe.2001.115461

[17] Bodner L. Cystic lesions of the jaws in children. Int J Pediatr Otorhinolaryngol. 2002;62(1):25–9. 11738690 10.1016/s0165-5876(01)00583-3

[18] Borrás-Ferreres J, Sánchez-Torres A, Gay-Escoda C. Malignant changes developing from odontogenic cysts: A systematic review. J Clin Exp Dent. 2016;8(5):e622–8. 27957281 10.4317/jced.53256 PMC5149102

[19] Yasuoka T, Yonemoto K, Kato Y, Tatematsu N. Squamous cell carcinoma arising in a dentigerous cyst. J Oral Maxillofac Surg. 2000;58(8):900–5. 10935592 10.1053/joms.2000.8219

[20] Jain M, Mittal S, Gupta DK. Primary intraosseous squamous cell carcinoma arising in odontogenic cysts: an insight in pathogenesis. J Oral Maxillofac Surg. 2013;71(1):e7–14. 23092745 10.1016/j.joms.2012.08.031

[21] Kumchai H, Champion AF, Gates JC. Carcinomatous transformation of odontogenic keratocyst and primary intraosseous carcinoma: a systematic review and report of a case. J Oral Maxillofac Surg. 2021;79(5):1081.e1–1081.e9. 10.1016/j.joms.2020.12.046 33529609

[22] Ye P, Wei T, Gao Y, Zhang W, Peng X. Primary intraosseous squamous cell carcinoma arising from an odontogenic keratocyst: case series and literature review. Med Oral Patol Oral Cir Bucal. 2021;26(1):e49–55. 33037806 10.4317/medoral.23947 PMC7806341

[23] Jalali E, Ferneini EM, Rengasamy K, Tadinada A. Squamous cell carcinoma arising within a maxillary odontogenic keratocyst: a rare occurrence. imaging science in dentistry. Korea (South). 2017;47:135–40. 10.5624/isd.2017.47.2.135. 10.5624/isd.2017.47.2.135 PMC5489670 28680851

[24] Makowski GJ, McGuff S, Van Sickels JE. Squamous cell carcinoma in a maxillary odontogenic keratocyst. J Oral Maxillofac Surg. 2001;59(1):76–80. 11152194 10.1053/joms.2001.19297

[25] Falaki F, Delavarian Z, Salehinejad J, Saghafi S. Squamous cell carcinoma arising from an odontogenic keratocyst: a case report. Med Oral Patol Oral Cir Bucal. 2009;14(4):E171–4. 19333185

[26] Sharif FN, Oliver R, Sweet C, Sharif MO. Interventions for the treatment of keratocystic odontogenic tumours (KCOT, odontogenic keratocysts (OKC)). Cochrane Database Syst Rev. 2010;(9):CD008464. 10.1002/14651858.cd008464.pub3. 10.1002/14651858.CD008464.pub2 20824879

[27] Slootweg PJ, Richardson M. Diagnostic surgical pathology of the head and neck. 2009.

[28] Suma NK, Babu NSV, Jha S, Dental V. In. Odontogenic keratocyst of maxillary premolar region: a case report. 2015. https://www.semanticscholar.org/paper/Odontogenic-Keratocyst-of-Maxillary-Premolar-A-Case-Suma-Babu/730b531de200ab391700cc34f6d329c3b6fa2837.

[29] Gardner AF. The odontogenic cyst as a potential carcinoma: A clinicopathologic appraisal. J Am Dent Assoc. 1969;78(4):746–55. 4887224 10.14219/jada.archive.1969.0290

[30] Yoshida H, Onizawa K, Yusa H. Squamous cell carcinoma arising in association with an orthokeratinized odontogenic keratocyst. Report of a case. J Oral Maxillofac Surg. 1996;54(5):647–51. 8632255 10.1016/s0278-2391(96)90653-9

[31] Deepa KK, Jannu A, Kulambi M, Shalini HS. A case of dentigerous cyst in a pediatric patient - With an insight into differential diagnostic entities. Adv Oral Maxillofacial Surg. 2021;3:100130.

[32] Mendes RA, Carvalho JFC, van der Waal I. Characterization and management of the keratocystic odontogenic tumor in relation to its histopathological and biological features. Oral Oncol. 2010;46(4):219–25. 20189443 10.1016/j.oraloncology.2010.01.012

[33] Chen P, Liu B, Wei B, Yu S. The clinicopathological features and treatments of odontogenic keratocysts. Am J Cancer Res. 2022;12(7):3479–85. 35968329 PMC9360231

[34] El-Naggar AK, Chan JKC, Rubin Grandis J, Slootweg PJ. WHO classification of head and neck tumours. 2017. 10.1007/s00428-018-2320-6.

[35] Bianco BCF, Sperandio FF, Hanemann JAC, Pereira AAC. New WHO odontogenic tumor classification: impact on prevalence in a population. J Appl Oral Sci. 2020;28:e20190067. 31778444 10.1590/1678-7757-2019-0067 PMC6882648

[36] Cavalcanti MGP, Veltrini VC, Ruprecht A, Vincent SD, Robinson RA. Squamous-cell carcinoma arising from an odontogenic cyst–the importance of computed tomography in the diagnosis of malignancy. Oral Surg Oral Med Oral Pathol Oral Radiol Endod. 2005;100(3):365–8. 16122667 10.1016/j.tripleo.2004.12.012

[37] Aboul-hosn Centenero S, Marí-Roig A, Piulachs-Clapera P, Juárez-Escalona I, Monner-Diéguez A, Díaz-Carandell A, et al. Primary intraosseous carcinoma and odontogenic cyst. Three new cases and review of the literature. Med Oral Patol Oral Cir Bucal. 2006;11(1):E61–5. 16388297

[38] Mosqueda Taylor A, Meneses García A, Ruíz Godoy Rivera LM, de Suárez Roa M. Luna Ortiz K. Malignant odontogenic tumors. A retrospective and collaborative study of seven cases. Med Oral. 2003;8(2):110–21. 12618671

[39] Scheer M, Koch AMAM, Drebber U, Kübler ACAC. Primary intraosseous carcinoma of the jaws arising from an odontogenic cyst–a case report. J Craniomaxillofac Surg. 2004;32(3):166–9. 15113575 10.1016/j.jcms.2003.12.005

[40] Chaisuparat R, Coletti D, Kolokythas A, Ord RA, Nikitakis NG. Primary intraosseous odontogenic carcinoma arising in an odontogenic cyst or de novo: a clinicopathologic study of six new cases. Oral Surg Oral Med Oral Pathol Oral Radiol Endod. 2006;101(2):194–200. 16448921 10.1016/j.tripleo.2005.03.037

[41] Medawela RMSHB, Jayasuriya NSS, Ratnayake DRDL, Attygalla AM, Siriwardena BSMS. Squamous cell carcinoma arising from a keratocystic odontogenic tumor: a case report. J Med Case Rep. 2017;11(1):335. Available from: 10.1186/s13256-017-1486-x. 10.1186/s13256-017-1486-x PMC5710129 29191248

[42] Hantash AA, Shuibat AM, Çobanoğlu N, Yıldrım B, İçten O, Senguven B. PP160: right hemi-maxillectomy via Weber Ferguson approach for the management of squamous cell carcinoma of the maxilla and paranasal sinuses. Oral Oncol. 2013;49:S148.

[43] Menon S. Keratocystic odontogenic tumours: Etiology, pathogenesis and treatment revisited. J Maxillofac Oral Surg. 2015;14(3):541–7. 26225042 10.1007/s12663-014-0734-5 PMC4511900

